# Seed amplification assay of nasal swab extracts for accurate and non-invasive molecular diagnosis of neurodegenerative diseases

**DOI:** 10.1186/s40035-023-00345-1

**Published:** 2023-03-16

**Authors:** Suying Duan, Jing Yang, Zheqing Cui, Jiaqi Li, Honglin Zheng, Taiqi Zhao, Yanpeng Yuan, Yutao Liu, Lu Zhao, Yangyang Wang, Haiyang Luo, Yuming Xu

**Affiliations:** 1 Department of Neurology, The First Affiliated Hospital of Zhengzhou University, Zhengzhou University, Zhengzhou, China; 2 Department of Rhinology, The First Affiliated Hospital of Zhengzhou University, Zhengzhou, China; 3 The Academy of Medical Sciences of Zhengzhou University, Zhengzhou University, Zhengzhou, China; 4 Henan Key Laboratory of Cerebrovascular Diseases, The First Affiliated Hospital of Zhengzhou University, Zhengzhou University, Zhengzhou, China; 5 Institute of Neuroscience, Zhengzhou University, Zhengzhou, China

**Keywords:** Nasal swab, Seed amplification assay, Prion disease, Amyloid, Neurodegenerative diseases

## Abstract

Nasal swabs are non-invasive testing methods for detecting diseases by collecting samples from the nasal cavity or nasopharynx. Dysosmia is regarded as an early sign of coronavirus disease 2019 (COVID-19), and nasal swabs are the gold standard for the detection. By nasal swabs, severe acute respiratory syndrome coronavirus 2 (SARS-CoV-2) nucleic acids can be cyclically amplified and detected using real-time reverse transcriptase-polymerase chain reaction after sampling. Similarly, olfactory dysfunction precedes the onset of typical clinical manifestations by several years in prion diseases and other neurodegenerative diseases, such as Alzheimer’s disease, Parkinson’s disease, dementia with Lewy bodies, and multiple system atrophy. In neurodegenerative diseases, nasal swab tests are currently being explored using seed amplification assay (SAA) of pathogenic misfolded proteins, such as prion, α-synuclein, and tau. These misfolded proteins can serve as templates for the conformational change of other copies from the native form into the same misfolded form in a prion-like manner. SAA for misfolded prion-like proteins from nasal swab extracts has been developed, conceptually analogous to PCR, showing high sensitivity and specificity for molecular diagnosis of degenerative diseases even in the prodromal stage. Cyclic amplification assay of nasal swab extracts is an attractive and feasible method for accurate and non-invasive detection of trace amount of pathogenic substances for screening and diagnosis of neurodegenerative disease.

**Graphical Abstract:**

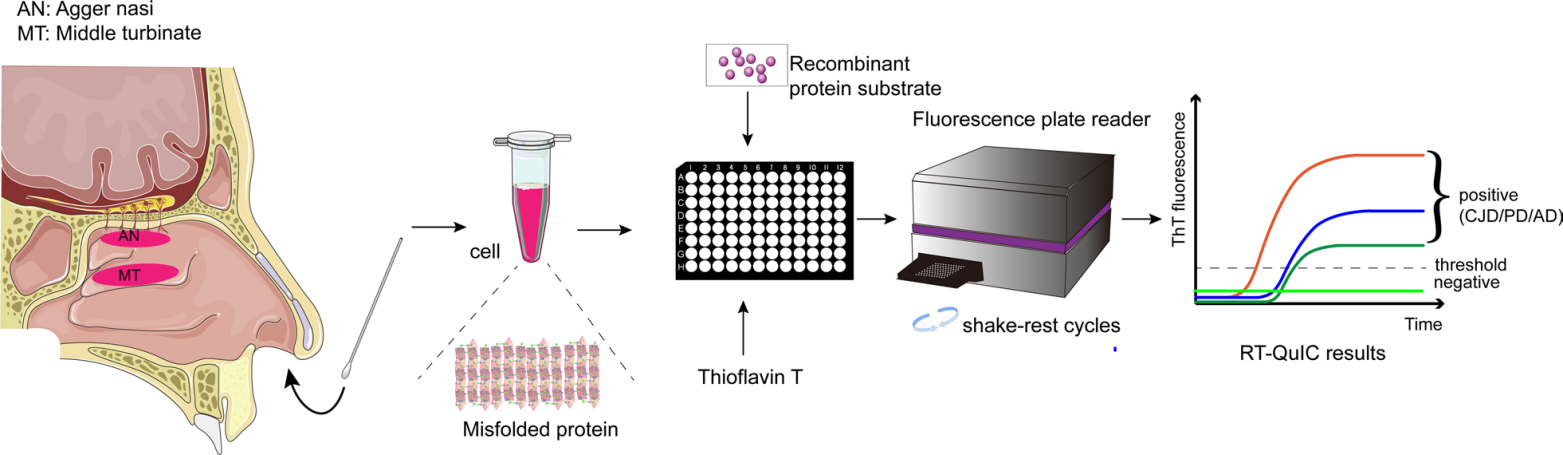

## Background

Neurodegenerative diseases are a heterogeneous group of disorders characterized by progressive degeneration of neuron structure or function, such as Alzheimer’s disease (AD), Parkinson’s disease (PD), amyotrophic lateral sclerosis (ALS), Huntington's disease (HD), and prion diseases [1]. Accurate diagnosis of a neurodegenerative disease is vital for the screening, diagnosis, and subsequent management of patients. However, a definite diagnosis of neurodegenerative diseases is tricky and requires brain biopsies or autopsies. These disorders are mainly diagnosed clinically [2]. Due to the complexity and heterogeneity of neurodegenerative disorders and a large number of overlapping clinical manifestations, an exact diagnosis is often challenging [3, 4]. Even experienced clinicians may misdiagnose them. A non-invasive and accurate strategy for the diagnosis of neurodegenerative diseases is warranted [5].

Nasal swabs are non-invasive testing methods for detecting diseases by collecting samples from the nasal cavity or nasopharynx. Olfactory dysfunction is an early sign of most neurodegenerative diseases [6, 7], and a growing body of research indicates that the olfactory pathway may be one of the initially affected areas in patients with neurodegenerative diseases [8–11]. Nasal swab tests, analogous to PCR, are currently being explored using seed amplification assays (SAA) of pathogenic misfolded proteins in neurodegenerative diseases.

Neurodegenerative diseases are characterized by the accumulation of disease-related misfolded proteins, such as α-synuclein, Aβ, and tau, which are known for their prion-like self-amplifying capacity. Protein-misfolding cyclic amplification (PMCA) and  real-time quaking-induced conversion (RT-QuIC) assays are legacy names from the prion field and we will use the name SAA to refer to the assay for non-prion proteins in the present review. SAA has been developed to diagnose neurodegenerative diseases in accessible biospecimens, such as cerebrospinal fluid (CSF) [12–38], skin [33, 34, 39–42], and olfactory mucosa [17, 26, 43–50]. SAA of nasal swab extracts is the most prominent for its simple, rapid and non-invasive sampling, and shows very high sensitivity and specificity in detecting neurodegenerative diseases, even at the prodromal stage [17].

In this review, we discuss the anatomy of the nasal cavity and olfactory system, summarize the sample collection and testing process of nasal swabs, and highlight the clinical application of nasal swabs in prion diseases, synucleinopathies and tauopathies, focusing on the early and non-invasive diagnosis of neurodegenerative diseases via SAA of nasal swab extracts.

### Basic anatomy of olfaction and the olfactory pathway

**Fig. 1:**
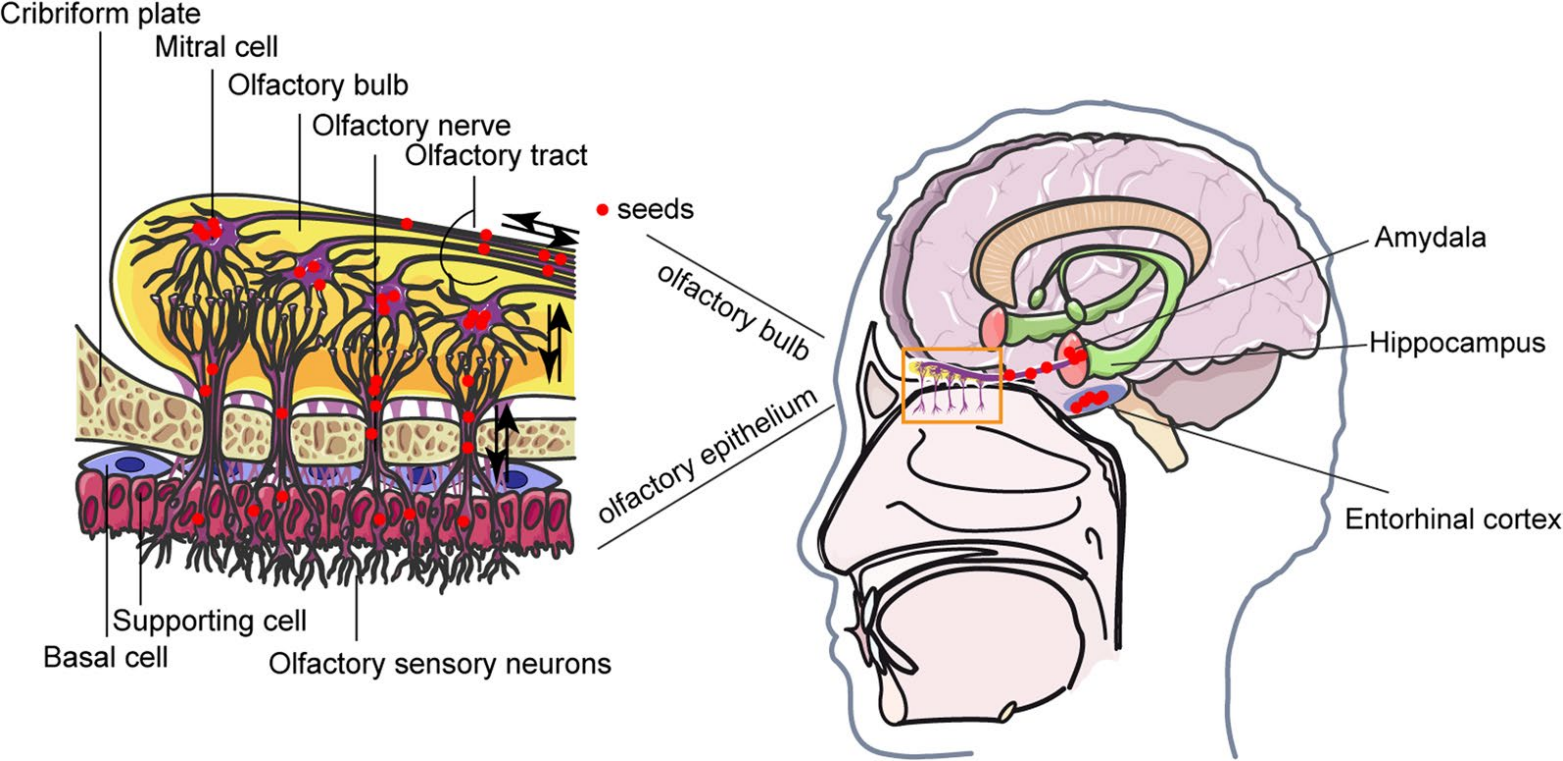
The olfactory conduction pathway. The olfactory epithelium comprises three cell types: olfactory sensory neurons (OSNs), supporting cells, and basal cells. After OSNs capture odor molecules, the axons of neurons expressing the same odorant receptors converge to several well-defined glomeruli in the olfactory bulb (OB), where they transmit signals to mitral cells. The axons of the mitral cells leave the OB and project to the higher olfactory cortex, including the piriform cortex, hippocampus, and amygdala, to generate the sense of smell. Misfolded protein aggregates transfer between the olfactory system and the central nervous system via olfactory pathways. The red dots represent seeds

The olfactory epithelium consists of three types of cells with different morphology and functions: olfactory sensory neurons (OSNs), supporting cells, and basal cells [51]. OSNs are bipolar neurons and exist in the mucosa of the upper nasal cavity. The dendritic processes of OSNs cross the mucosa at the top of the nasal cavity, the upper part of the nasal septum, and the medial part of the superior nasal nail to form olfactory receptors. The central processes of OSNs form the olfactory nerve, which transmits the olfactory impulse to the olfactory bulb. Basal cells can differentiate into OSNs [52]. The olfactory bulb is the first relay station of an olfactory pathway that transmits and processes olfactory information, where the central processes of OSNs form synapses with the dendrites of mitral and tufted cells. Then, the axons of mitral and tufted cells transmit olfactory information to the olfactory cortex through the olfactory tract [53] (Fig. 1).

### Distribution of aggregates in the nasal cavity

Misfolded protein aggregates commonly deposit in the olfactory system in patients with neurodegenerative diseases [54]. AD and PD, two most common neurodegenerative diseases, both exhibit olfactory dysfunction early in disease course despite different phenotypes and pathologies [6, 7]. Beach et al. found the presence of Lewy bodies in the olfactory bulb of patients with PD by immunohistochemical staining for α-synuclein [54]. Braak et al. proposed the pathological staging of PD, in which α-synuclein aggregates propagate from the olfactory bulb to the central nervous system [11, 55]. Consistent with observations in humans, animal studies injecting preformed α-synuclein fibrils into the olfactory bulb of mice have shown appearance of preformed α-synuclein fibrils in other brain regions after a period of time [56–59]. Ohm et al. reported that neurofibrillary tangles and neuropil threads occur in the anterior olfactory nucleus and olfactory bulb in AD cases [60]. This finding has been repeatedly confirmed, suggesting that this pathology starts very early [61–63]. Subsequently, Arnold et al. found tau aggregates in the olfactory epithelium of patients with AD [64]. In the early stages of AD and PD, the pathological involvement of anosmia and olfactory pathways has led to the hypothesis that AD and PD are caused by substances entering the brain through olfactory pathways [65]. However, so far, there is no direct evidence to support this hypothesis.

### Nasal swabs and SAA of nasal swab extracts

**Fig. 2:**
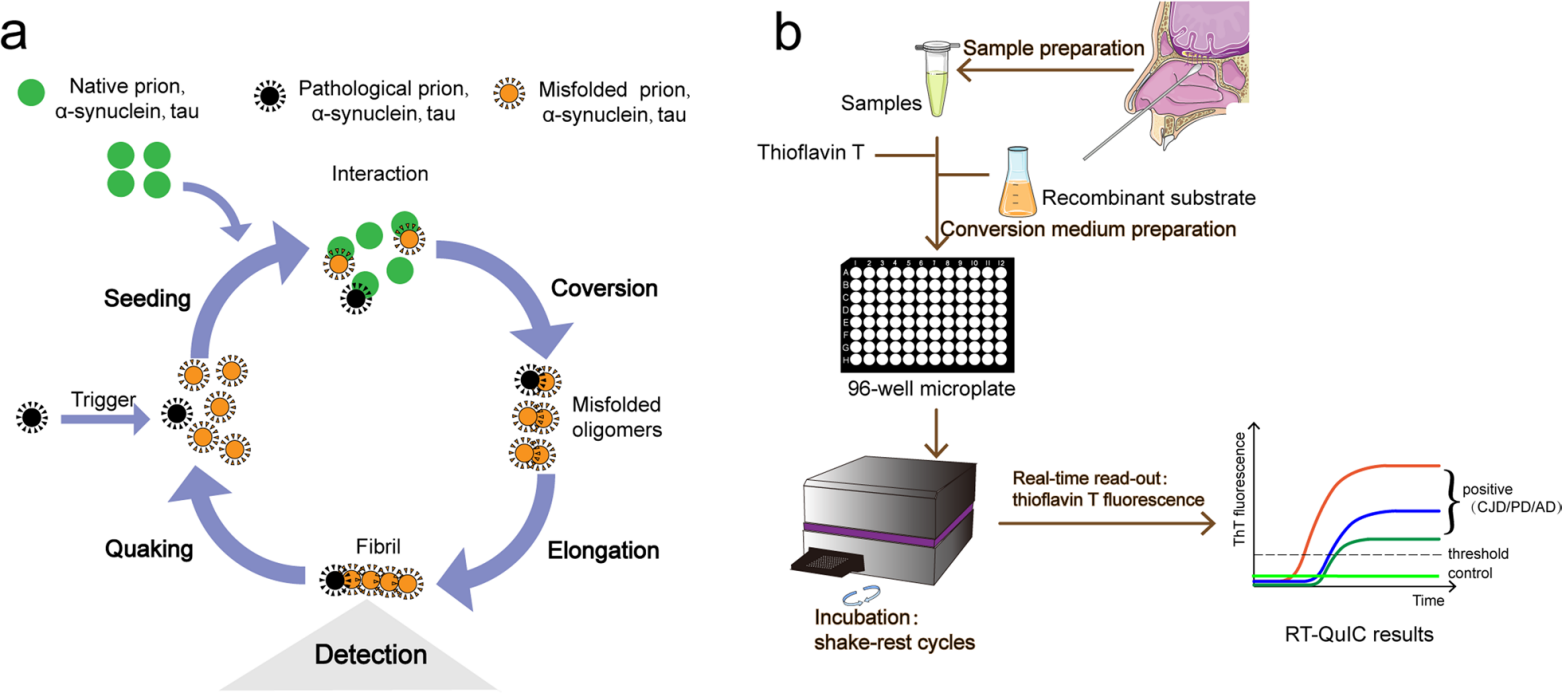
Schematic presentation of RT-QuIC mechanism and process of nasal swab extracts. **a** Seed transformation in the RT-QuIC process. The misfolded proteins (orange, seeds) trigger conformational changes of native proteins (green, substrates) to form new misfolded seeds. This switch results in conformational modification into misfolded oligomers, which are then elongated into fibrils. After detection of fibrils, longer fibrils are broken down into shorter reactive seeds by quaking, which further facilitate the conversion of native proteins. **b** RT-QuIC procedure using nasal swab extracts. Sample homogenate from the patient’s olfactory mucosa is mixed with the reaction buffer containing recombinant protein and Thioflavin T (ThT, a fluorescent amyloid probe), loaded into a multi-well microplate, and then incubated with alternating cycles of shaking and rest in a fluorescence microplate reader. This device records ThT fluorescence emission throughout the incubation, thus revealing the kinetics of aggregate formation. Sigmoidal growth in ThT fluorescence indicates a positive diagnosis

Nasal swabs are a non-invasive testing method that detects diseases by collecting samples from the nasal cavity or nasopharynx. Currently, nasal swabs are being used to diagnose neurodegenerative diseases via collection of affected cells in the olfactory mucosa, from which misfolded proteins are detected. The misfolded proteins can serve as a template for proteins of the same type to misfold. The SAA technology was first described under the name PMCA [66] and later modified to use recombinant protein (named rPrP-PMCA) [67], shaking (named QuIC) [68], and finally thioflavin T (ThT) readings (final name RT-QuIC) [13]. SAA is a representative technique to amplify trace amounts of misfolded protein aggregates in tissues and biofluids to detect the seeding activity of pathological proteins, in the aim of diagnosing neurodegenerative diseases [13, 69]. Misfolded proteins aggregate into oligomers that are further elongated into fibrils detected using ThT fluorescence. Shaking breaks down the fibrils into shorter oligomers (seeds), which can combine with other natural proteins to facilitate transformation and continuous cyclic amplification (Fig. 2) [70]. Thus, SAA is based on cyclic amplification, which is different from other detection methods such as cell culture and antigen detection.

Nasal swab sampling from patients with neurodegenerative diseases is performed at the olfactory mucosa, and the sampling method is slightly complicated and relatively non-invasive. The position of the olfactory mucosa is first located using a nasal scope and then nasal swab sampling is performed [17, 71]. A detailed tutorial video on olfactory mucous sampling is accessible at https://www.youtube.com/watch?v=wYb9W3u6uMY [26].

### Application of nasal swabs in neurodegenerative diseases

Similar to COVID-19, olfactory dysfunction is an early symptom of most neurodegenerative diseases. Olfactory dysfunction is related to the deposition of pathological proteins, such as misfolded α-synuclein and tau protein, identified in a post-mortem study [72]. In recent years, researchers have combined nasal swabs with SAA to diagnose neurodegenerative diseases, and reported high sensitivity and specificity. The main results of performance of SAA in olfactory mucosa samples in diagnosing different neurodegenerative diseases are summarized in Table 1.

**Table 1:** Performance of seed amplification assays of olfactory mucosa samples in different neurodegenerative diseases

Disease	Number of cases	Number of controls	Sensitivity (%)		Specificity (%)	References
CJD	31	43	97		100	[17]
CJD	69	17	94		100	[26]
FFI	2	26	100		100	[43]
PD	18	18	56		17	[44]
MSA	11	18	82		17	[44]
CJD	9	19	100		100	[45]
CJD	35	7	91		100	[46]
PD	13	11	69		100	[47]
MSA-P	20	11	90		100	[47]
MSA-C	10	11	0		100	[47]
RBD	63	59	44.4		89.8	[48]
PD	41	59	46.3		89.8	[48]
PD	43	29	74		90	[49]
CJD	29	34	79.3		100	[50]
PSP	4	0	50		–	[93]
CBD	2	0	50		–	[93]
AD	2	0	0		–	[93]

*CJD* Creutzfeldt–Jakob disease, *FFI* Fatal Familial Insomnia, *PD* Parkinson's disease, *MSA* multiple system atrophy, *MSA-P* MSA patients with the parkinsonian phenotype, *MSA-C* MSA patients with the cerebellar phenotype, *RBD* Isolated REM sleep behaviour disorder, *PSP* Progressive supranuclear palsy, *CBD* Corticobasal degeneration, *AD* Alzheimer’s disease

#### Application of nasal swabs in prion diseases

Prion diseases are a group of progressive, incurable, and fatal neurodegenerative diseases that can affect both humans and animals. The infectious agent causing prion disease is known as the scrapie isoform of the prion protein (PrP^Sc^). Prion diseases are related to PrP^Sc^ accumulation in the central nervous system, caused by the autocatalytic conversion of normal cellular prion protein (PrP^c^) to repeated misfolded isoforms. Confirmation of the diagnosis of Creutzfeldt–Jakob disease (CJD) requires the detection of PrP^Sc^ in biopsy specimens; however, this method poses a risk to healthcare workers and also is invasive for patients [73].

In 2001, Saborio et al. reported a procedure involving cyclic amplification of protein misfolding that allows rapid conversion of large excess PrP^C^ into a protease-resistant, PrP^Sc^-like form in the presence of minute quantities of PrP^Sc^ template, a process called PMCA [66]. In 2007, Atarashi et al. used recombinant hamster PrP^C^ to replace brain-derived PrP^C^, which greatly accelerated the rate of seed polymerization and facilitated the development of rapid, ultrasensitive prion assays and diagnostic tests. This method is called rPrP-PMCA [67]. In 2008, Atarashi et al. developed a new prion assay, abbreviated QuIC for quaking-induced conversion, which uses automated tube shaking rather than sonication. This assay is faster and simpler than the PMCA and rPrP-PMCA assays [68]. In 2011, Atarashi et al. further improved the rapidity and practicality of this method by combining it with ThT fluorescence to monitor amyloid fibril formation. This assay is called RT-QuIC. They evaluated the technique in a blinded study of CSF samples in patients with CJD, achieving over 80% sensitivity and 100% specificity [13]. Later, the CSF RT-QuIC technique was increasingly used to diagnose CJD, with diagnostic sensitivity and specificity ranging 77%–100% and 98%–100%, respectively [14, 17, 19, 20, 74]. In view of the high sensitivity and specificity, CSF RT-QuIC has been included in the diagnostic criteria for CJD [75, 76]. Additionally, PMCA has been described for the detection of CJD in blood [77, 78] and urine [79].

Based on the existence of PrP^Sc^ in the olfactory neuroepithelium [80], olfactory mucosal sampling provides another promising strategy for the diagnosis of CJD. Compared with CSF, the collection of olfactory mucosa samples is simple, rapid, and non-invasive. In 2014, Orrú et al. used RT-QuIC to detect PrP^Sc^ in the olfactory mucosa of patients with CJD, with sensitivity and specificity of 97% and 100%, respectively, while testing CSF samples from the same group had a sensitivity of 77% and specificity of 100%. The olfactory mucosa can elicit a faster and stronger RT-QuIC response than the CSF [17]. Subsequently, Bongianni et al. combined results from RT-QuIC assays of CSF and olfactory mucosa samples to achieve an antemortem diagnosis of sporadic CJD with 100% specificity and sensitivity [26]. Fiorini et al. also showed that the combination of CSF and olfactory mucosa RT-QuIC testing led to 100% sensitivity and specificity, proving that it is feasible to include RT-QuIC detection of target proteins from CSF and olfactory mucosa samples in the diagnostic criteria of CJD [46]. And then, Orrú et al. developed "second-generation" RT-QuIC assays to detect PrP^Sc^ in the olfactory mucosa of CJD patients, with 100% sensitivity and 100% specificity [45]. In addition, Cazzaniga et al. used the PMCA technology to detect prions in the olfactory mucosa of CJD patients with 79.3% sensitivity and 100% specificity [50]. Fatal familial insomnia (FFI) is a genetic prion disease caused by a point mutation in the prion protein gene (*PRNP*). In 2017, Redaelli et al. demonstrated that the olfactory mucosa of patients with FFI contains PrP^Sc^ detectable by PMCA and RT-QuIC [43].

#### Application of nasal swabs in other neurodegenerative diseases

A growing body of evidence supports that the pathogenesis of neurodegenerative diseases is caused by the misfolding, aggregation, and spread of disease-associated proteins, as observed in prion diseases. Some of these proteins include misfolded α-synuclein in synucleinopathies and tau in tauopathies [81].

### Application of nasal swabs in synucleinopathies

Synucleinopathies are a group of diseases caused by misfolding and aggregation of α-synuclein, including PD, dementia with Lewy bodies (DLB), and multiple system atrophy (MSA) [82]. Compelling evidence suggests that in synucleinopathies, abnormally folded α-synuclein proteins are present in trace amounts in the CSF and peripheral tissues, such as the olfactory mucosa [83]. In 2016, Fairfoul et al. first developed a novel SAA assay for the detection of α-synuclein in the CSF of patients with DLB and PD, with sensitivities of 92% and 95%, respectively, and an overall specificity of 100% [84]. To date, researchers from various countries have used the SAA technology to detect α-synuclein in the CSF of patients with synucleinopathies, and obtained similarly high sensitivity and specificity [29, 85, 86].

Olfactory dysfunction is considered one of the earliest symptoms of PD, at least 4 years preceding classic motor deficits [6]. As in PD, olfactory dysfunction is common in DLB [87]. Rey et al. found that the olfactory bulb may be an entry site for prion-like transmission in neurodegenerative diseases [88]. The success of nasal swab SAA in diagnosing prion diseases promoted the application of nasal swab SAA in synucleinopathies. In 2019, De Luca et al. first applied the SAA technique to detect the seeding activity of α-synuclein in the olfactory mucosa of patients with PD and MSA, and reported a sensitivity of 55.6% and 81.8%, respectively, and an overall specificity of 83.3% [44].

More importantly, olfactory mucosa samples from patients with PD or MSA induce the formation of α-synuclein aggregates with different biochemical and structural characteristics, which is promising for differentiating between the two diseases. In detail, α-synuclein SAA products seeded with the olfactory mucosa of patients with MSA show stronger proteinase K resistance than the SAA products seeded with the olfactory mucosa of PD patients. In addition, the α-synuclein SAA products seeded with the olfactory mucosa of patients with PD and MSA also differ in structure. As observed with transmission electron microscopy, the distance between overtwists in α-synuclein fibrils acquired from SAA seeded with the MSA olfactory mucosa is greatly different from that acquired from SAA seeded with the PD olfactory mucosa [44]. These studies using nasal swabs are supported by similar studies in CSF samples where the different structures produced by PD and MSA are studied in more details [89].

Moreover, α-synuclein SAA with olfactory mucosa samples can not only be used to identify PD and MSA but also to identify different subtypes of MSA. MSA is an adult-onset sporadic neurodegenerative disease, which mainly includes two types: the parkinsonian (MSA-P) and the cerebellar (MSA-C) [90]. Bargar et al. found that efficient α-synuclein SAA seeding activity could be observed in the olfactory mucosa of MSA-P patients but not of MSA-C patients. The lack of α-synuclein seeding activity in MSA-C patients indicates that MSA-P and MSA-C may be caused by different strains of α-synuclein with different affinities to the olfactory mucosa [47].

Additionally, Perra et al. found that the consistency between SAA and clinical diagnoses was 86.4% for the olfactory mucosa and 93.8% for the CSF of patients with DLB. Interestingly, the research team performed a “dual-tissue α-synuclein SAA test” on the same patient, first testing the olfactory mucosa and then the CSF. The combined SAA detection of olfactory mucosa and CSF improved the consistency with clinical diagnosis to 100%. Perra et al. proposed a novel diagnostic approach in which the non-invasive nasal swabs can be used as a first-line screening procedure for patients with suspected DLB, and CSF analysis can be performed as a confirmatory test when the results of the olfactory mucosa are inconsistent with the initial clinical diagnosis [91].

Isolated rapid eye movement sleep behavior disorder (iRBD) and pure autonomic failure (PAF) are currently recognized to be prodromal for synucleinopathies [92, 93]. In 2020, Rossi et al. first used SAA to detect α-synuclein seeding activity in the CSF of patients with iRBD and PAF, and reported sensitivity of 100% and 92.9%, respectively. The specificity was 98% in 101 negative controls [32]. In 2021, Iranzo et al. reported similarly high sensitivity and specificity for SAA in detecting α-synuclein in the CSF of iRBD patients [37]. Subsequently, Stefani et al. analyzed olfactory mucosa samples from 63 patients with iRBD and 41 patients with PD in a blinded manner by α-synuclein SAA, and the sensitivity was 44.4% and 46.3%, respectively, but the specificity for iRBD plus PD versus controls was high (89.8%). The different sensitivities and specificities obtained in different studies could be largely due to the distinct SAA protocols used, which may outweigh the intrinsic differences on the samples. These results suggest that nasal swabs are attractive non-invasive tests for screening patients in the early stages of synucleinopathies [48].

Plenty of studies have conducted SAA analysis of α-synuclein using samples from the olfactory mucosa of PD, MSA and DLB patients. While the relative diagnostic accuracy for MSA and DLB was 81.8% and 86.4%, respectively in these studies, the relative diagnostic accuracy for PD was low, ranging from 46.3% to 55.6% [44, 48, 91]. In PD, the accuracy of SAA detection of pathological α-synuclein in the olfactory mucosa is inferior to that in other synucleinopathies. To investigate whether this is related to the distribution of pathological α-synuclein in the olfactory mucosa, Bongianni et al. performed nasal swab sampling in different areas covered by olfactory neuroepithelium, such as the agger nasi (AN) and middle turbinate (MT), and then performed SAA detection of α-synuclein. Two cohorts were analyzed in this study, and the results showed that the sensitivity of α-synuclein SAA in AN and MT were 78%–84% and 43%–45%, respectively. Subsequently, the nasal swab samples from PD patients were subjected to immunocytochemistry with an antibody for β-tubulin III, a phenotypic marker of olfactory neurons and their precursors. Immunofluorescence showed that the β-tubulin III-positive cells were more abundant in AN than in MT, consistent with the SAA results. These results reveal a new mechanism, where the deposition of abnormal α-synuclein in PD may preferentially occur in the AN and eventually spread to the entire olfactory mucosa. Consistent with previous findings in MSA, combined testing for CSF and nasal swab samples from PD patients increased the diagnostic accuracy to nearly 100% [49].

### Application of nasal swabs in tauopathies

Tauopathies are a group of heterogeneous diseases characterized by intracellular deposition of abnormally folded forms of the microtubule-associated protein tau. Tauopathies include Pick’s disease (PiD), corticobasal degeneration (CBD), progressive supranuclear palsy (PSP), and AD [10]. Several ultrasensitive cell-free tau SAAs have been developed to preferentially detect tau aggregates in the post-mortem brain tissues and CSF of patients with PiD, PSP, CBD and AD [25, 94–96]. However, these studies are limited in that the samples were derived from autopsy. The use of SAA in human antemortem diagnosis will largely depend on the ability to detect tau seeds in accessible tissues, such as the olfactory mucosa [96].

AD is the most common neurodegenerative disease leading to dementia. The symptoms of AD may be similar to those of other dementias, which can lead to misdiagnosis [97]. Approximately 85% of patients with AD develop anosmia in the early stage, before cognitive impairments appear [98]. Previous studies have shown that abnormal tau proteins can accumulate in the olfactory epithelium of patients with AD [64, 99]. Rossi et al. analyzed olfactory mucosa samples collected from several tauopathies, and reported that tau seeding activity was observed in some olfactory mucosa samples from PSP and CBD patients, but not in samples from AD patients. The detection may be influenced by substrate proteins, as PSP and CBD are 4R tauopathies and AD is 3R/4R tauopathy. This is in accordance with the results from previous studies, which showed poor tau seeding in AD under 3R tau SAA conditions[25] and 4R tau SAA conditions [94]. “3R + τ306” [95] or “K12” (residues 244–275 and 306–400 of the full-length human tau sequence) [96] tau SAA has been developed for brain homogenates and CSF for detection of AD. Thus, with the current available data, it is not possible to say whether these results reflect biological differences between diseases or just technical problems to detect different isoforms of tau seeds. The tau-SAA technique needs to be further optimized to effectively detect tau seeding activity in biological samples (e.g., CSF and olfactory mucosa) collected from living patients.

Despite moderate overall sensitivity, nasal swab SAA is attractive and more easily accepted by patients as a simple, fast, and non-invasive approach compared with other specimen detection methods. Nasal swab SAA could be considered as a first-line screening procedure in patients suspected of neurodegenerative diseases. Further research is required to enhance the detection sensitivity and understand the role of the olfactory mucosa in neurodegenerative diseases.

## Conclusions and future directions

The SAAs in nasal swab extracts are simple, rapid, and non-invasive. When compared with lumbar puncture, nasal swabs can be performed in people taking anticoagulants. The olfactory mucosa can elicit a fast and strong SAA response with 97% sensitivity and 100% specificity in prion diseases. SAAs in nasal swab extracts from patients with PD, DLB and MSA show high sensitivity and specificity and could also detect α-synuclein seeding activity in prodromal-stage synucleinopathies, such as iRBD and PAF. Moreover, α-synuclein SAA in the olfactory mucosa could be used to distinguish between PD and MSA by discriminating α-synuclein strains. The sensitivity of nasal swab SAA may be improved by adjusting the sampling site, which needs to be confirmed by further studies.

Anosmia is also common in other neurodegenerative diseases, such as ALS, frontotemporal dementia (FTD), and Huntington’s disease, which also involve prion-like misfolded proteins. Although not mentioned above, the SAA assay can detect TDP-43 seeds. The pathological deposition of TDP-43 occurs in most cases (~ 97%) of ALS and in approximately 45% of FTD cases. Scialò et al. used SAA to detect TDP-43 seeding activity in the CSF of patients with ALS and FTD, with an overall sensitivity and specificity of 94% and 85%, respectively [10]. Research is urgently needed to identify the sensitivity of nasal swab SAA in detecting these diseases. In addition, Kurihara et al. demonstrated that high-field magnetic resonance imaging and diffusion tensor tractography can be used to visualize olfactory sensory neurons, and further development of this technique may advance its clinical use for the diagnosis of olfactory dysfunction [100].

In summary, SAA in nasal swab extracts is a simple, fast, and non-invasive method that is more easily accepted by patients compared with other specimen detection methods. Combined testing of the olfactory mucosa and other samples can improve the diagnostic accuracy. Nasal swabbing can be considered as a first-line screening procedure in patients suspected of neurodegenerative diseases, followed by CSF detection as a confirmatory test when the results of olfactory mucosa SAA are inconsistent with the clinical diagnosis. Further neuropathological studies are crucial for understanding the pathology initiation of prion-like proteins in the olfactory mucosa and the role of the olfactory pathway in the development of neurodegenerative diseases.

## Data Availability

Not applicable.

## Abbreviations

AD: Alzheimer’s disease
ALS: Amyotrophic lateral sclerosis
AN: Agger nasi
CBD: Orticobasal degeneration
CJD: Creutzfeldt–Jakob disease
COVID-19: Coronavirus disease 2019
CSF: Cerebrospinal fluid
DLB: Dementia with Lewy bodies
FTD: Frontotemporal dementia
iRBD: Isolated rapid eye movement sleep behavior disorder
MSA: Multiple system atrophy
MT: Middle turbinate
OSN: Olfactory sensory neuron
PAF: Pure autonomic failure
PD: Parkinson’s disease
PiD: Pick’s disease
PrP^c^: Normal cellular prion protein
PrP^Sc^: Scrapie isoform of the prion protein
PSP: Progressive supranuclear palsy
SAA: Seed amplification assay
SARS-CoV-2: Severe acute respiratory syndrome coronavirus 2
ThT: Thioflavin T
